# Cross-cultural adaptation and clinical validation of the Neonatal Skin Condition Score to Brazilian Portuguese^1^

**DOI:** 10.1590/0104-1169.3456.2487

**Published:** 2014

**Authors:** Juliana Machado Schardosim, Luma Maiara Ruschel, Giordana de Cássia Pinheiro da Motta, Maria Luzia Chollopetz da Cunha

**Affiliations:** 2 Doctoral student, Universidade de Brasília, Brasília, DF, Brazil. Assistant Professor, Universidade de Brasília, Brasília, DF, Brazil; 3 Intern in Nursing, Hospital de Clínicas de Porto Alegre, Porto Alegre, RS, Brazil; 4 MSc, RN, Hospital de Clínicas de Porto Alegre, Porto Alegre, RS, Brazil; 5 PhD, Associate Professor, Escola de Enfermagem, Universidade Federal do Rio Grande do Sul, Porto Alegre, RS, Brazil

**Keywords:** Validation Studies, Dermatology, Skin Care, Newborn, Nursing

## Abstract

**OBJECTIVE:**

to describe the process of cross-cultural adaptation and clinical validation of the Neonatal Skin Condition Score.

**METHODS:**

this methodological cross-cultural adaptation study included five steps: initial translation, synthesis of the initial translation, back translation, review by an Committee of Specialists and testing of the pre-final version, and an observational cross-sectional study with analysis of the psychometric properties using the Adjusted Kappa, Intraclass Correlation Coefficient, and Bland-Altman Method statistical tests. A total of 38 professionals were randomly recruited to review the clarity of the adapted instrument, and 47 newborns hospitalized in the Neonatology Unit of the Clinical Hospital of Porto Alegre were selected by convenience for the clinical validation of the instrument.

**RESULTS:**

the adapted scale showed approximately 85% clarity. The statistical tests showed moderate to strong intra and interobserver item to item reliability and from strong to very strong in the total score, with a variation of less than 2 points among the scores assigned by the nurses to the patients.

**CONCLUSIONS:**

the scale was adapted and validated to Brazilian Portuguese. The psychometric properties of the Brazilian version of the Neonatal Skin Condition Score instrument were similar to the validation results of the original scale.

## Introduction

Birth is the principle and most rapid change of environment. The infant needs to adapt in the transition between the aquatic environment of the womb, with a constant temperature, into our environment, with characteristically lower humidity and other variations^(^ 1 ^)^. The skin is of vital importance during this period. Its primary function is the barrier against pathogenic microorganisms and topical substances (toxic or not), and it is also involved in the maintenance of thermal and hydro-electrolytic balance^(^ 2 ^-^ 3 ^)^.

Maintaining healthy skin is essential for newborns (NBs), especially for premature and full-term NBs hospitalized in the neonatal unit^(^ 2 ^-^ 3 ^)^. The hospitalized babies are constantly exposed to invasive procedures, disinfectant substances, use of adhesives for fixing devices, as well as to nosocomial bacterial flora, therefore they usually present more obvious alterations in the skin surface than non-hospitalized babies^(^ 2 ^)^.

The evaluation of the skin conditions of hospitalized neonates constitutes part of the daily physical examination and needs to be frequent and objective. With this aim, the Neonatal Skin Condition Score (NSCS) was published in 2004 in the United States. The NSCS was validated in a large study investigating 51 Neonatal Skin Care institutions. The study was started in 1997 by the Association of Women´s Health Obstetric and Neonatal Nurses - AWHONN, together with the National Association of Neonatal Nurses NANN, mainly aiming for the development of an Evidence-Based Clinical Practice Guideline - Neonatal Skin Care. This is a short instrument of rapid application that can be included in the Brazilian healthcare practice to support the neonatology healthcare teams^(^ 2 ^,^ 4 ^-^ 5 ^)^. The NSCS evaluates three factors: *dryness, erythema,* and *breakdown*. Each item has three possible answers giving a score from 1 to 3. The final score of the patient is obtained by summing the responses of the three items, ranging from 3 to 9, 3 being the best condition and 9 the worst skin condition that the NB could have^(^ 4 ^-^ 5 ^)^.

The use of scales in the care practice serves to standardize the evaluation of the health status of patients and nursing interventions through care protocols. The use of these scales in other countries with cultures and languages different from those in which the scales were created depends on the scientific rigor with which they are translated, evaluated and revised in the language for which they are to be used, therefore multicultural studies have gained ground in the same way as the use of instruments for standardization of the healthcare in different cultures^(^ 6 ^)^.

The development of the study is justified due to its contribution to the knowledge of nursing professionals about skin fragility of NBs, considering the repercussions of skin lesions in this population as well as the use of scales in the clinical practice. Due to the anatomy and important functions of the skin during the adaptation of the NB, the preservation of skin integrity represents an important nursing care action, being even more relevant in the neonatal period. Therefore, it is believed that the use of this instrument in the clinical practice assists in standardizing the nurses' actions and in improving the neonatology care quality.

The NSCS has not been validated for the Portuguese language of Brazil, therefore interest in validating the instrument for Brazil emerged, aiming to facilitate and standardize the evaluations of the professionals and, subsequently, their interventions. The aim of the study was to perform the cross-cultural adaptation and clinical validation for the use of the Neonatal Skin Condition Score instrument in Brazil.

## Methods

Two distinct steps were used to perform the study: a) cross-cultural adaptation, which consisted of the translation and adaption of the instrument for Brazilian Portuguese based on the methodological procedures proposed by Beaton et al.^(^ 7 ^)^; b) clinical validation, which consisted of the application by professionals of the final Portuguese version in the clinical practice, aiming to verify the psychometric properties through an observational cross-sectional study.

During the cross-cultural adaptation, the semantic (each item maintaining the same meaning after the translation into another language), idiomatic (searching for expressions or corresponding explanations in the target language, as idioms can not be translated), conceptual (verifying that the different concepts used in the different cultures have the same connotation) and experimental (assessing whether the terms used in the instrument are appropriate for the clinical practice in the culture of the language into which the scale is being validated) equivalences were analyzed to avoid distortions from one language to the other^(^ 7 ^)^. This same process has been used in the validation of other instruments for use in Brazil^(^ 8 ^-^ 11 ^)^.

The cross-cultural adaptation is performed in five stages: initial translation, synthesis of the translations, back translation, evaluation by an Committee of Specialists, and testing of the pre-final version^(^ 7 ^)^.

The initial translation consisted of the translation of the original instrument from English to Brazilian Portuguese, carried out by two translators with different technical profiles (one with and one without knowledge in the healthcare area), both of who's native language was Brazilian Portuguese. Each translator produced one independent version relative to one another, in a blinded way^(^ 7 ^)^.

In the synthesis of the translations a technical review and evaluation of equivalence of the versions of the initial translation was carried out by a language professional and the translators. At the end of this step a consensus version of the initial translations was produced^(^ 7 ^)^.

In the back-translation the consensus version was re-translated from Portuguese back to English by two translators who's native language was English and who had no technical training in the healthcare area. The translators were blinded in relation to each other and to the original instrument. In this step a consensus of the back-translated versions was produced and this consensus version was sent to the author of the original version of the NSCS, in order to compare the back-translated version with the original version.

The evaluation by a committee of specialists was conducted through a meeting to review all the versions produced, in order to reach the pre-final version of the instrument in Brazilian Portuguese. The committee was composed of 1 teacher with experience in the method of cross-cultural adaptation, 1 language professional, 1 dermatology specialist nurse, and the translators^(^ 7 ^)^.

Testing of the pre-final version was carried out in the final phase of the cross-cultural adaptation process and aimed to evaluate the clarity of the items that compose the instrument^(^ 7 ^)^. A total of 38 professionals (physicians, nurses, auxiliary nurses and nursing technicians) of the Neonatal Hospitalization Unit of the Clinical Hospital of Porto Alegre (UIN/HCPA) were selected at random with the aid of a random number table^(^ 12 ^)^. Each professional recorded his impressions about the clarity of the pre-final Portuguese version of the instrument using a Likert type scale^(^ 12 ^)^. The Likert scale responses were numbered from 1 to 5 with 1 being "not at all clear" and 5 "totally clear". In addition to responding to the instrument, the participants recorded suggestions and justifications of their responses in a specific space for comments.

With the finalization of the adaptation process, the clinical validation of the adapted version of the NSCS was initiated. The population included NBs hospitalized in the UIN/HCPA, who were included in the study on the first day of hospitalization. Only those NBs from other hospitals that did not have all the data surveyed in their patient records were excluded from the study. Using the Stata version 7. 0 program, the sample calculation was 36 NBs assuming a Kappa ≥ 0.4 (0.1;0.7) and a 95% confidence interval for an error of 0.3 (in the 0.5% CI). The sample selection was by convenience.

The integrity of the skin of each NB was evaluated 4 times (2 in person and 2 through digital images), using the Portuguese version of the NSCS, by 2 nurses in a blinded way. Digital images were obtained at the time of the in person evaluation, to avoid change in skin condition between the in person evaluations and digital images, and were evaluated by the nurses approximately 10 days after the in person evaluation to ensure that the memory of the nurse did not interfere in the score of the infant. In this way it was possible to evaluate the intraobserver reliability, which represents the stability of the scale in the evaluation of the skin condition of the same patient by the same examiner, and the interobserver reliability, which represents the stability of the scale regarding evaluations made by different professionals related to the skin condition of the same patient^(^ 13 ^)^.

The internal consistency of the instrument could not be verified because the items of the NSCS are independent, i.e., one does not influence the value of the other. The concurrent criterion validity could not be verified due to the lack of other skin evaluation instruments for newborns that could be used as a gold standard^(^ 12 ^-^ 13 ^)^.

The statistical analysis of the study was performed using the Statistical Package for the Social Sciences (SPSS) and from web based calculators for SCR analysis. The clarity of the instrument was verified by means of descriptive statistics with results expressed as absolute and relative frequencies of the percentage of clarity by summing the concepts (clear, very clear, and totally clear) of each item evaluated. The analysis of the total score of the Likert scale was also performed, this result was expressed as the mean and standard deviation of the sum of responses of the items evaluated in the Likert scale^(^ 12 ^)^.

A descriptive analysis of the demographic data of the study sample was performed. Categorical variables were expressed as absolute and relative frequencies, whereas the continuous variables were expressed as mean and standard deviation when symmetric and as median and 25 and 75% percentiles when asymmetric.

For the evaluation of the psychometric properties of the Portuguese version of the instrument, the following statistical tests were used: Adjusted Kappa (PABAK), which evaluated the intra and interobserver variability of each item that comprised the scale, as these are categorical variables, the intraclass correlation coefficient (ICC) and the Bland-Altman method, which analyzed the intra and interobserver reliability of the total score of the instrument, as these are continuous variables. A significance level of 5% (α =0.05) was considered^(^ 13 ^-^ 15 ^)^.

The copyright of the instrument of this study is owned by the Association of Women's Health, Obstetric and Neonatal Nurses (AWHONN), therefore, formal permission was obtained to conduct the study and publish the article. The project was approved by the Research Ethics Committee of the Clinical Hospital of Porto Alegre under No. 110344. Resolution 466/12, regarding the regulatory guidelines involving research with humans, was followed and the professionals and patients invited to take part in the study signed the Terms of Free Prior Informed Consent^(^ 16 ^)^.

## Results

**Figure 1:**
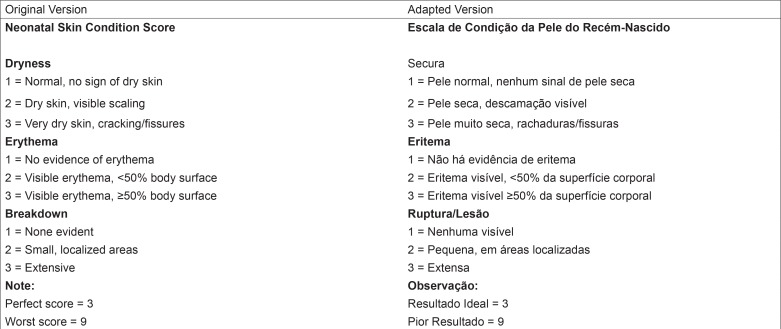
- **Table presenting the original version of the NSCS and the version adapted for Brazilian Portuguese** Neonatal Skin Care Evidence-Based Clinical Practice Guideline Third Edition (Appendix A), by Association of Women's Health. Obstetric and Neonatal Nurses, 2013, Washington, DC: Association of Women's Health, Obstetric and Neonatal Nurses. Copyright (2013) by the Association of Women's Health, Obstetric and Neonatal Nurses. Adapted with permission.

The cross-cultural adaptation sought to translate and adapt the NSCS for Brazilian Portuguese, aiming to correct the technical terms used in accordance with the semantic, idiomatic, experimental and conceptual equivalence. The original version and the version adapted to Brazilian Portuguese of the NSCS are presented in Figure 1.

The title and the terms *dryness, breakdown *and *note* demanded greater attention and a search for the concepts in the literature in the English and Portuguese languages by presenting different words for translation. The author of the original instrument participated in the translation and adaptation process of the instrument with clarification of the concepts of the technical terms in English. In addition, the consensus version of the back translation was evaluated to verify the possible differences between the back-translation consensus version and the original version. Accordingly, the answer obtained was that there were no distortions to be corrected. 

After the corrections, the pre-final version of the scale in Portuguese underwent the Pre-Final Version Testing step. The recruited sample of 38 professionals consisted of 6 physicians (15.8%), 14 nurses (36.8%) and 18 auxiliary nurses/nursing technicians (47.4%). The result of the analog scale (Likert) verified the clarity of the instrument, demonstrated in absolute and relative frequencies. The sum of the clear, very clear, and totally clear responses was considered as clear, observing clarity of 85% in the majority of the items. Thus it was found that the scale was clear and easily understood by the healthcare professionals.

Clinical validation was carried out between May and July 2012, the final sample consisted of 47 NBs with no losses during the data collection. The demographic profile of the study sample is shown below (Table 1).

**Table 1:** - Demographic profile of the study sample. Porto Alegre, RS, Brazil, 2012

		n (%)
Gender (Male)		25 (53.2)
Birth weight (grams)*		2685.64 (934.03)
Apgar No. 1^st^/5^st^ minutes of life*		7.28 (2.7)/ 8.83 (1.2)
Type of birth (vaginal)		27 (57.4)
Gestational age (weeks)*		37 (3)
Age at hospitalization (days of life)^†^		1(1-2)
Reason for Hospitalization in Neonatology Unit		
	Early Respiratory Dysfunction	11 (23.4)
	Prematurity	10 (21.3)
	Jaundice	7 (14.9)
	Other	19 (40.4)

* Symmetric continuous variables expressed as mean ± standard deviation

† Asymmetric continuous variables expressed as median (25% - 75% percentiles)

Analysis of the reproducibility and intraobserver and interobserver reliabilities was tested through three simultaneous tests: the PABAK, the ICC and the Bland-Altman tests, as shown in Table 2 ^(^ 12 ^,^ 14 ^-^ 15 ^)^.

**Table 2:** - Intra and interobserver reliability of the final Portuguese version of the NSCS. Porto Alegre, RS, Brazil, 2012

		Neonatal Hospitalization Unit		
		n=47		
		Concordance	p	Magnitude
Intraobserver				
	Dryness*	77.2% (0.66)	<0.001	Strong
	Erythema*	76.1% (0.66)	<0.001	Strong
	Rupture/Injury*	85.9% (0.79)	<0.001	Strong
	Total score^†^	0.88	<0.001	Very Strong
Interobserver				
	Dryness*	69.6% (0.54)	<0.001	Moderate
	Erythema*	70.7 (0.56)	<0.001	Moderate
	Rupture/Injury*	81.5 (0.72)	<0.001	Strong
	Total score^†^	0.61	<0.001	Strong

* Concordance expressed as a percentage of concordance and adjusted Kappa values

† Concordance calculated through the Intraclass Correlation Coefficient

The Bland-Altman method was a test complementary to the ICC and verified the variation between the responses of the nurse evaluators. While the ICC verifies the correlation between the responses of the evaluators, the Bland-Altman method evaluates the variation between the compared scores starting from a graphical view (scatter chart) that presents the bias and trends of the responses analyzed. The bias, presented in the charts, represents how much the differences between the scores moved away from zero^(^ 14 ^)^, i.e., the mean variation between the compared scores. The upper correlation limit (UCL) and the lower correlation limit (LCL) values are the upper and lower limits of the differences in the score of the patient among the evaluators (interobserver) and between the two evaluations performed by the same observer with each patient (intraobserver)^(^ 14 ^)^.

Dispersion was observed in the responses of the evaluators in both the inter and intraobserver comparisons. The results showed a variation in the interobserver comparison of up to 1.38 points more (UCL) and 1.87 points less (LCL) with a bias of 0.24, while there was a variation of 0.83 points more (UCL) and 1.03 points less (LCL), with a bias of 0.01 in the intraobserver comparison. It should be noted that, even with variations of up to almost 2 points in the evaluations of the skin condition of patients, the variation median (bias) was small. These findings corroborate the results of the other tests, demonstrating a strong correlation between the compared final scores.

## Discussion

The choice of the NSCS as the study object of this work was due to the importance of standardizing the daily evaluation of the skin integrity to detect early changes in the skin of NBs^(^ 4 ^-^ 5 ^)^. This instrument is the only one published in the literature that can evaluate the skin conditions of NBs, although another instrument is able to evaluate the risk and occurrence of pressure ulcers in children (Braden Q Scale)^(^ 8 ^)^. The use of the NSCS can only be observed in studies published in the English language, which leads us to believe that this is the first translation of the instrument into another language^(^ 17 ^-^ 18 ^)^.

The use of health scales is widespread. Differences between definitions exist that require translation and cultural adaptation prior to the use of an instrument in a language or culture different to that for which it was prepared, in order to achieve the best possible translation through a meticulous and accurate method with constant verification of the equivalences already described in the method section of this paper. 

Historically, the use of instruments created in another culture or language was limited to a simple translation of the original version to the new language in which the scale would be used, however, it is believed that the simple translation resulted in instruments that differed from the original due to the personal understanding of the translator. Cultural adaptation turned the translation into a scientific method, producing a result more faithful to the original and consistent with the new culture in which the instrument is to be used^(^ 19 ^)^. 

Another important advantage of the cross-cultural adaptation process is the back-translation, as this enables discussions between the authors of the original version and the translated version, favoring the exchange of knowledge and the resolution of doubts and uncertainties arising from the translation process. In this study, the author of the original instrument actively participated throughout the translation and adaptation process of the instrument with clarification of the concepts of the technical terms in English^(^ 10 ^)^.

The evaluation of clarity at the end of the cultural adaptation phase was crucial for the final alterations prior to carrying out the clinical trial. Some adjustments were also performed from the analysis of the percentages of clarity and from the subjective observations recorded on the forms completed by the healthcare professionals. The percentages of clarity from the evaluation using the Likert scale were considered satisfactory because it was a heterogeneous sample of professionals with different levels of knowledge and different amounts of professional experience. This phase is included in the adaptation process, therefore it is not described in the article covering the validation of the original version of the instrument^(^ 4 ^)^.

Upon completion of the cross-cultural adaptation process the final version in Portuguese of the NSCS called *Escala de Condição da Pele do Recém-Nascido *(ECPRN) was tested with its application in the practice by a team of nurses. Comparing the correlation between the psychometric properties of the NSCS and the ECPRN it was observed that the PABAK values found in the Brazilian version were higher. The intraobserver ICC in the Brazilian validation was more significant than in the validation of the original version of the NSCS, while the interobserver ICC was very similar^(^ 4 ^)^.

Even with moderate magnitude in some items of the scale, calculated by the PABAK, in the final score of the scale the magnitudes were strong to very strong. This is probably due to the fact that the scale is composed of 3 independent items. The same trend was noticed in the validation study of the original version^(^ 4 ^)^, and these data show that even when an item is evaluated differently by nurses ultimately the skin condition score of the patient is often the same as when it is evaluated item by item, thus demonstrating stability in the scale.

From the clinical point of view, a better correlation in the total score is more important because in the daily practice this is the score that guides professionals in decision making regarding whether to intervene or not in the skin condition of the infant. Correlation coefficients are considered acceptable from 0.6, which represents a strong correlation, to 1, which represents a perfect correlation^(^ 13 ^)^.

The Bland-Altman method demonstrated that there was variability in the intra and interobserver scores. The greater score variation observed in the intraobserver comparison may be due to the use of digital images, as this is an comparison of the evaluation of the same patient in person and by digital images. It is understood that the ambient lighting conditions, technical conditions of the machine, and movements of the NB while obtaining the digital images may have affected the quality of the image and therefore the total skin score assigned to the patient. Retesting by digital images rather than in person was considered a limitation of the study, however, the decision to use these images was due to the fact that the skin undergoes changes throughout the adaptation process of the NB and the need to compare the scores assigned by the nurse with another evaluation conducted by the same nurse on the same patient for the validation of the instrument^(^ 1 ^,^ 5 ^)^.

## Final considerations

It was concluded that the Brazilian version of the instrument, called *Escala de Condição da Pele do Recém-Nascido* (ECPRN), was adapted and validated for use in Brazilian Portuguese. After completion of the translation and cultural adaptation process the phase to phase analysis performed showed good results. The clarity of the instrument was verified with healthcare professionals in the final step of the process in order to confirm the findings obtained in the previous steps by the study group.

The results of the analysis of the psychometric properties showed that the scale in Portuguese can be easily applied in neonatology nursing care in Brazil. It could serve as a tool for the evaluation of skin care in neonatology in the Brazilian reality, aiding in the improvement of NB skin care practices. It is suggested that future intervention studies with skin care for the newborn are performed using the ECPRN as an evaluation tool.
